# Report on Proceedings of the Eighth Annual European CME Forum, Manchester, UK, November 2015

**DOI:** 10.3402/jecme.v5.31705

**Published:** 2016-04-25

**Authors:** Ron Murray

**Affiliations:** ^a^ Independent CME/CPD Consultant, Pickering, UK

**Keywords:** CME, CPD, activities, learners, accreditation, inter-professional, commercial support

## Abstract

Delegates from Europe, Asia, and North America attended the Eighth European CME Forum in Manchester, UK, on 12 and 13 November 2015. A new format that included three separate workshop tracks was introduced. The workshops focused on standards and accreditation, education and partnerships, funding and practice in CME/CPD. Discussion and interactive sessions on accreditation issues, inter-professional education, backwards planning, and patient engagement were among the topics addressed.

Facilitated conversations were conducted with European leaders in the medical profession and a representative from a European commercial supporters’ organisation. Panel discussions on current and future trends and the views of local junior doctors representing the learner community were also conducted.

A reinvention of the 8th Annual European CME Forum to facilitate the coalescence of people, organisations, and ideas in the style of an ancient Roman forum was reflected in its new venue – the city of Manchester in northwest England, included in Lonely Planet's list of top ten global cities due to its “transformation in recent years, with inspirational new openings, the reinvention of existing spaces and vast investment.”

A diverse group of delegates from Europe, Asia, and North America met at the Radisson Blu Hotel in Manchester on 12 and 13 November 2015 to participate in interactive workshops and discussions that covered a range of topics including:

1. Activity planning
2. Accreditation and regulation
3. Inter-professional education
4. Industry support
5. Patient engagement
6. Facilitated conversations with international leaders and learners in CME/CPD

Those in attendance represented the spectrum of medical education providers, accreditation organisations, commercial supporters, and academic institutions. The dialogue was sometimes provocative, sometimes harmonious, but consistently engaging.

The 8th Annual Forum commenced with Eugene Pozniak's review of the previous year's developments in European CME including the Forum's new website (www.europeancmeforum.eu) and the collaboration between the *Journal of European CME* (JECME) and the *Journal of Continuing Education in the Health Professions* (JCEHP) to provide a greater spread of publishing opportunities for CME researchers and practitioners. It was also noted that the measurement of outcomes continued to be the most important topic.

The opening session served as an ice-breaker when Alisa Pearlstone and Celeste Kolanko of PCM Scientific guided the Forum delegates through a table top exercise to produce a list of 20–30 elements necessary to plan and implement a “standalone” accredited CME activity in Europe. The elements suggested were then compared with a list of 24 produced by the Good CME Practice Group (www.goodcmepractice.net) and the exercise continued with delegate tables being tasked with providing an appropriate sequence for the suggested elements.

The rest of Day 1 followed the new workshop format with a choice of workshops available based on three separate tracks:

1. Standards and accreditation – the Star Track
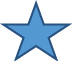

2. Education and partnerships – the Circle Track
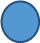

3. Funding and practice – the Triangle Track
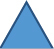

The three Star Track workshops comprised presentations by representatives from European, US, and Canadian accrediting bodies.

1. Edwin Borman and Nathalie Paulus representing UEMS-EACCME provided practical tips on new criteria that guide the application process for accreditation of international CME activities outside the United States and Canada and indicated that streamlining the process was a current goal of UEMS-EACCME.
2. The US presenter, Kate Regnier of ACCME examined a range of methods for determining practice-based gaps and educational needs and how such needs might be addressed through various activity formats, conforming to the accreditation criteria set by ACCME.
3. Jennifer Gordon of the Royal College of Physicians and Surgeons of Canada expanded the perspective to a global level to consider common values, principles, and metrics of accreditation systems around the world. She highlighted the content of the Consensus Statement from the Second International Forum on CPD Accreditation, held on 19 and 20 March, 2010, in Sydney, Australia, as well as the role of the recently formed International Academy for CPD Accreditation.

Circle Track

1. Three US presenters Don Moore (Vanderbilt University), Kathy Chappell (American Nurses Credentialing Centre), and Lawrence Sherman (TOPEC Global) were joined by London physician Mathena Pavan (University College London Hospital) to explore the use of a combined model of an Outcomes Framework and Instructional Design to conduct a backwards planning exercise in producing a CME activity. Participants were encouraged to develop an action plan for use in implementing the combined model.
2. Kathy Chappell teamed up with Jann Balmer (University of Virginia) to engage participants in a case study via role play and group discussion to further illustrate the outcomes framework in the design of an inter-professional education solution to a specific health care “problem in practice.”
3. Laura Muttini (Educational Health Strategies, LLC) moderated a workshop session that considered global trends in patient education/engagement and their growing inclusion in CPD programming. This was neatly complemented with examples of proven techniques of engagement including gamification and social support systems. A real-life example was provided with input from a patient involved in a local educational initiative on Rheumatoid Arthritis.

Triangle Track.

1. Jann Balmer and Laura Muttini were joined by Lisa Sullivan from Australia to represent the Global Alliance for Medical Education (GAME) with a competitive game along the lines of The Amazing Race. Using clues to illustrate differences among regulations in various European countries, participants came up with suggested strategies to implement solutions for a range of health issues within the confines of those countries’ regulations. The competitive atmosphere was enhanced by the fact that the winning team received complimentary registration to the 2016 GAME Conference in Barcelona, Spain.
2. Maureen Doyle-Scharff (Pfizer, International Pharmaceutical Alliance for CME) described her company's Global Independent Grants Program and led discussion of industry (commercial) support under the new umbrella of transparency in CME funding and provided a comparison of US and European approaches.
3. A European perspective was provided by Marian East (MedSense, UK) and Diana van Brakel (Kenes Education, Amsterdam) in their presentation of case studies to illustrate issues associated with the need to educate faculty members and CME planners about the requirements for independence and a robust evidence-based approach to planning and implementing European CME activities. Their points were emphasised by showing that there should be no difference in the approach to implementing a European CME-accredited conference supported by 15 companies for 8,000 delegates or one supported by one company for 200 delegates.

Day 2 continued with the Forum's growing tradition of conversational interviews with European representatives of academia, a physician specialty organisation, and an industry membership federation.

1. Robin Stevenson (Editor-in-Chief, JECME) and Ian Bruce (Professor of Rheumatology, University of Manchester) discussed DevoManc – a scheme whereby the Health and Social Care budget will be devolved to local control in Greater Manchester. A pertinent point of discussion was looking at the possibility of establishing a CME “department” for Greater Manchester.
2. Craig Campbell (RSPSC) discussed with Reinhard Griebenow (ECSF) transparency in CME and the changing role of CME providers given the lack of over-riding legislation in Europe for CME accreditation.
3. Marie-Claire Pickaert (EFPIA) discussed with Maureen Doyle-Scharff (Pfizer) the EFPIA Transparency Code and issues of country-specific privacy laws.

The Forum continued with panel discussions on current CME practice and future trends, skilfully moderated by UK health journalist Jacqui Thornton

The first “value of CME” session explored current practice with Vassilios Papalois (Secretary General-elect, UEMS), Mark Westwood (St Bartholomew's Hospital), and Alistair Thomson (Consultant Paediatrician, Associate Postgraduate Dean, Health Education NorthWest (HENW)). Points that emerged from the discussion include:

1. No real reflection on practice-based needs for CPD in Europe
2. Language barriers
3. Problems still exist in defining direct and indirect funding

From a “What the future holds” session with Edwin Borman (Secretary General, UEMS), Craig Campbell (Director, Professional Affairs, Royal College of Physician and Surgeons of Canada), Marie-Claire Pickaert (EFPIA), and Peter Mills (Barts Heart Centre, European Cardiology Section Foundation Board Member), emerging points were:

- The need for consultants to realise the importance of ongoing learning.
- Learners need to differentiate between “branded” promotional education and truly independent medical education.
- There is a need to move away from one-size education and make the learner less anonymous.

The panel discussions were interspersed with a lunchtime question and answer session with a panel of local junior physicians who described their experiences and feelings about the CME activities available to them. One point made by this panel that raised some eyebrows among the delegates was the fact that none of these physicians had ever been involved in providing needs assessment information for any of their CME activities.

The almost traditional “unsession” led by global CME presenter Lawrence Sherman closed the Forum and allowed the delegates to tie up loose ends such as a discussion on payment by industry for “travel grants” and some suggestions for workshop topics for the 2017 ECF in Amsterdam.

Full details of the presentations and support materials may be accessed at the European CME Forum website: www.europeancmeforum.eu

The Twitter stream for the meeting can also be obtained at www.europeancmeforum.eu/wp-content/uploads/2015/06/8ECF-Twitter-Stream.pdf

## Conflict of interest and funding

The author has not received any funding or benefits from industry or elsewhere to conduct this study.

